# Does Familial Domestic Abuse Present Differently to Intimate Partner Violence? An Exploratory Analysis of Police Data Examining Parent-, Child-, and Sibling-Perpetrated Domestic Abuse

**DOI:** 10.3390/bs16101811

**Published:** 2026-10-03

**Authors:** Louise Almond, Paige Monaghan, Michelle McManus, Danielle Murphy, Lauren Halsall, Jo-Pei Tan

**Affiliations:** 1 Department of Psychology, University of Liverpool, Eleanor Rathbone Building, Liverpool L69 7ZA, UK; 2 School of Nursing and Public Health, Manchester Metropolitan University, Brooks Building, Manchester M15 6GX, UK; p.monaghan@mmu.ac.uk (P.M.); m.mcmanus@mmu.ac.uk (M.M.); 3 Division of Health Research, Faculty of Health and Medicine, Lancaster University, Health Innovation One Building, Lancaster LA1 4AT, UK; l.halsall@lancaster.ac.uk; 4 Department of Social Care and Social Work, Manchester Metropolitan University, Ormond Building, Manchester M15 6BX, UK; j.tan@mmu.ac.uk

**Keywords:** DA, policing, risk-assessment, DASH, DARA, familial DA

## Abstract

Domestic Abuse (DA) cases perpetrated by family members account for a substantial proportion of reported DA cases, yet responses within policing and support services remain largely informed by evidence derived from Intimate Partner Violence (IPV). Although familial DA includes a range of family relationships, including parent, child, sibling, it remains unclear whether these cases present differently to IPV and therefore require different responses. This study examined routinely collected police data from 122,276 DA incidents reported to a UK police force between April 2019 and March 2023. Perpetrator, victim, offence, risk, and case outcome characteristics were examined for parent-, child-, and sibling-perpetrated DA, with each subgroup compared separately with cases of IPV. Statistically significant differences were identified across all familial perpetrator groups; however, the magnitude and pattern of these differences varied considerably. When compared with the IPV subsample child-perpetrated DA demonstrated the greatest differentiation across perpetrator, victim, offence, and risk characteristics, whereas parent- and sibling-perpetrated DA showed comparatively modest divergence. These findings suggest familial DA should not be treated as a single category and that current DA responses, including the routine use of IPV-informed risk assessments for non-IPV cases, may not be equally appropriate across all forms of familial DA.

## 1. Introduction

The *Domestic Abuse Act* (2021) defines Domestic Abuse (DA) as any abusive, violent, threatening, controlling or coercive behaviour between personally connected individuals aged 16 years or over. In the year ending March 2024, an estimated 2.3 million people experienced DA in England and Wales, with Intimate Partner Violence (IPV) representing the most commonly recognised form (Office for National Statistics, 2024). However, national estimates suggest that over one third of DA cases involve abuse from a family member rather than an intimate partner (Office for National Statistics, 2024). Despite its prevalence, familial DA has received considerably less research attention than IPV and remains comparatively underrepresented within both the evidence base and policing practice.

Familial DA encompasses a range of perpetrator–victim relationships, including abuse perpetrated by parents, children and siblings. These relationships differ in their social and familial dynamics and may therefore present different patterns of offending, victimisation and risk. Therefore, familial DA is not a homogenous crime but rather encompasses a range of abuse associated with unique circumstances and challenges. Sibling abuse, for example, is often minimized due to societal norms around sibling rivalry or arguments (Caffaro, 2026; Kettrey & Emery, 2006). Alternatively, parents who have experienced abuse from their children often internalize feelings of parental failure, guilt and shame (Cottrell, 2001), and may even face judgment and ostracism from professional support services due to this societal stigma (Klun et al., 2025; Brule & Eckstein, 2016; S. Holt, 2011). These issues may also be compounded by the perpetrators’ legal and social reliance on the victim, acting as a further barrier to speaking out (Brule & Eckstein, 2016). Child-to-parent abuse may also encompass elder abuse when the victim is aged over 60. In such cases, the victim may be dependent on the perpetrator for care and support (Teaster et al., 2026). Furthermore, a recent systematic review by Klun et al. (2025) argues that violence perpetrated by adult children against their parents is not recognised by society as readily as other forms of family violence. Consequently, in order to appropriately target responses to DA and most effectively support all types of DA victims, it may be beneficial to consider the circumstances surrounding the abuse, including the relationship between the victim and perpetrator.

Despite these differences, current policing responses to DA remain largely informed by evidence derived from IPV. In England and Wales, the DA, Stalking, Harassment and Honour-Based Violence (DASH) risk assessment is routinely used across DA cases to assess risk and inform safeguarding decisions (Richards, 2009). However, DASH was developed primarily within an IPV context and is based upon risk factors that may not fully capture the circumstances surrounding other forms of DA (McManus et al., 2017; Turner et al., 2022) and thus may not be suitable for all cases. Indeed, concerns regarding the predictive validity of DASH have already been raised, with research demonstrating that the tool continuously fails to identify the most vulnerable victims and may contribute to high levels of victim withdrawal, calling instead for more evidenced-based, suspect-focused tools (Almond et al., 2017; McManus & Phoenix, 2023; Turner et al., 2019, 2022). Notably, the failure of the DASH tool may be particularly relevant in cases of familial DA, where the likelihood of victim withdrawal is potentially furthered by fear of disrupting the family environment (Burnes et al., 2019; A. Holt, 2016) and risk factors have limited ability to predict child to parent DA recidivism (McManus et al., 2017), but the tool nevertheless continues to be used in these instances. Consequently, it remains unclear whether applying a single risk assessment approach across all forms of DA is appropriate.

These issues are also relevant as police forces increasingly implement newer approaches such as the Domestic Abuse Risk Assessment (DARA), which was developed for first responding officers and places greater emphasis on coercive control and professional judgement (College of Policing, 2022). Evaluating newer tools across different DA relationship contexts is therefore important, rather than assuming that moving from DASH to DARA will automatically resolve the challenges associated with applying IPV-informed approaches to familial DA.

Beyond risk assessment strategies, many existing support services for both victims and perpetrators also fail to account for the heterogenous nature of DA, and recent policing reports have identified a need for further successful intervention (Vulnerability and Policing Futures Research Centre, 2025). Critically, as the academic literature around DA is predominated by feminist perspectives of IPV, perpetrator interventions are often specifically targeted at male, intimate-partner perpetrators and therefore less effective for other perpetrator cohorts (Barner & Carney, 2011; Laskey, 2016; Zhang & Howard, 2020). Likewise, support services and shelters are commonly designed for females who are victims of IPV (Moore, 2021), and therefore may not be inclusive to familial DA victims. Resultingly, alongside the development of suspect-focused risk assessment tools, more targeted support services are needed.

To support the development of evidence-based policing and public health responses, a growing body of literature has begun to investigate familial DA, with a particular focus on the role of experiencing abuse in childhood. Research consistently evidences previous experiences of abuse as a risk factor for perpetration of family violence (Goddard & Bedi, 2010; Hattery & Smith, 2026; Herrenkohl, 2011). From a theoretical perspective, this cycle is often conceptualized as a product of social learning, whereby abuse experienced or witnessed in childhood is learned, normalized, and subsequently carried out (Ehrensaft et al., 2003; Hattery & Smith, 2026; Wagner et al., 2019). Alternatively, the relationship between experiencing and subsequently perpetrating familial abuse may be psycho-biologically or cognitively determined, with altered stress responses and maladaptive information processing both proposed to play central roles (Caffaro, 2026; Finkelhor et al., 2013; Hattery & Smith, 2026; Herrenkohl et al., 2022; Teaster et al., 2026; Wolfe et al., 2001). Together, these findings suggest that familial DA may arise through a range of pathways, which may in turn contribute to variation in offending behaviour across different family relationships.

Early maladaptive schemas may provide one way of understanding this cognitive pathway. These schemas refer to enduring patterns of thinking and feeling about the self, others, and close relationships that can develop following harmful or unstable early experiences. Childhood abuse and neglect have been associated with adolescent maladaptive schemas (May et al., 2022), and research on child-to-parent aggression suggests that schemas and social information processing may help explain how young people interpret conflict, threat, rejection, or aggression within family relationships (Orue et al., 2021).

Research has also begun to identify characteristics associated with specific forms of familial DA. Across cases of child-to-parent DA specifically, perpetrators are most likely to be male/biological sons and to commit acts of abuse where other children are present (McManus et al., 2017), with both ineffective educational styles and peer violence identified as additional risk factors (Junco-Guerrero et al., 2025). In contrast, parent-to-child abuse is consistently associated with perpetrator mental health (Augusti et al., 2021; David-Ferdon, 2021; Patwardhan et al., 2017) and victim social isolation and health issues (Klun et al., 2025). More broadly, a comparison of IPV and non-IPV DA perpetrators found that non-IPV perpetrators are often younger and more likely to re-offend, potentially due to lower participation in interventions. They may also be more likely to perpetrate physical abuse (Zhang & Howard, 2020).

Despite these advances, important gaps remain. Research into DA-perpetrator characteristics and risk factors still predominantly focuses on IPV (Zhang & Howard, 2020), and much of the literature investigating familial violence explores cases of abuse not meeting the criteria for DA (e.g., forms of family violence that fall outside the legal definition of DA, such as abuse involving younger children). Furthermore, familial DA is often explored in the context of ‘non-IPV’ and thus complexities around specific family relationships are not investigated, with some types of familial DA such as sibling violence remaining highly under-researched (Elliott et al., 2020). As a result, little is known about whether different forms of familial DA present similarly to IPV, or whether some familial relationship types differ sufficiently to question the appropriateness of current IPV-informed risk assessment and policing responses.

### The Current Study

Current approaches to DA risk assessment and policing are largely informed by evidence derived from IPV, yet it remains unclear whether different forms of familial DA present in similar ways to IPV, or whether the nature of any differences varies across familiar relationship types. Understanding whether familial perpetrator relationships differ from IPV is important in determining whether existing approaches, including the routine use of the DASH risk assessment, are appropriate across all forms of DA. The current study therefore examined perpetrator, victim, offence, risk and case outcome characteristics across three forms of familial DA: parent-, child-, and sibling-perpetrated abuse. Each subgroup was compared with IPV to determine the extent to which they differed from the profile upon which current DA responses are largely based. The study addressed the following research questions:

(1) Do cases of parent-, child-, and sibling-perpetrated DA differ in perpetrator, victim, offence, risk and case outcome characteristics from IPV?
(2) To what extent do any observed differences vary across familial perpetrator relationships?

## 2. Materials and Methods

### 2.1. Sample

The current study used cross-sectional, routinely collected data on DA crimes and incidents reported to a UK Police force between April 2019 and March 2023. DA was defined in accordance with the *Domestic Abuse Act* (2021); therefore, only cases involving victims and perpetrators aged 16 years or over were included. Following data cleaning and pre-processing, the final dataset comprised 122,276 DA cases. The unit of analysis was the police-recorded DA incident/crime rather than the unique perpetrator, victim, or perpetrator-victim dyad.

### 2.2. Variables

#### 2.2.1. Perpetrator Characteristics

Perpetrator age, gender (male, female, or other), and relationship to the victim (parent, child, sibling, other family member, or current/former intimate partner) were extracted from police records.

#### 2.2.2. Victim Characteristics

Victim age, gender (male, female, or other), and ethnicity (White, Black, Asian, Mixed, or Other) were extracted from police records.

#### 2.2.3. Offence Characteristics

##### Primary Offence

The primary offence was categorised as sexual, violence against the person with injury, violence against the person without injury, violence against the person: stalking and harassment, theft, arson and criminal damage, or other.

##### Risk Assessment

Risk was categorised using the DASH tool (Richards, 2009). Overall risk level was recorded as standard, medium, or high. In addition, individual DASH risk factors were extracted as binary variables (present/absent), including repeat DA, perpetrator intoxication with alcohol, child risk, intimidation, online crime, and perpetrator mental health concerns.

#### 2.2.4. Case Outcome

The case outcome was categorised as charged, resolved out of court, evidential difficulties, victim does not support, no suspect identified, or other.

### 2.3. Data Analysis

Data were analysed using IBM SPSS Statistics (Version 29) NY, USA. Descriptive statistics were first calculated to summarise perpetrator, victim, offence, risk, and case outcome characteristics.

To examine whether familial DA differed from IPV, each familial perpetrator subgroup (parent-, child-, and sibling-perpetrated DA) was compared separately with IPV.

Chi-square tests of independence were conducted to determine whether the distribution of perpetrator, victim, offence, DASH risk, and case outcome variables differed between each familial subgroup and IPV. Given the large sample size, even very small differences are likely to be statistically significant. Therefore, Cramér’s V was calculated for all statistically significant associations to assess the magnitude of these differences consistent with established guidance on large-sample inference (Sullivan & Feinn, 2012; Lantz, 2013).

Cramér’s V was interpreted using Cohen’s (1988) guidelines, whereby values of 0.10, 0.30 and 0.50 represent small, medium and large effect sizes, respectively. Differences in perpetrator and victim age were examined using Mann–Whitney U tests due to the non-normal distribution of age, with *r* used as the corresponding effect size. In interpreting the bivariate findings, greater emphasis was placed on the size and practical meaning of the observed differences rather than statistical significance alone. Findings with *V* or *r* below 0.10 were interpreted cautiously and were only retained for discussion where they showed a clear relative difference between groups, were supported by the corresponding regression model, or appeared consistently across the familial DA subgroups Given the number of bivariate comparisons conducted for each subgroup, a Bonferroni-adjusted significance threshold of *p* < 0.003 was applied to the chi-square and Mann–Whitney U analyses to reduce the likelihood of Type I error.

Missing data were not imputed. For descriptive and bivariate analyses, valid cases were used for each variable, meaning that percentages and tests were calculated using the available non-missing data for the relevant variable. For the logistic regression models, SPSS applied listwise deletion for the variables entered into each model. Variables with substantial missingness, including ethnicity, primary offence type, and case outcome, were retained in the descriptive and bivariate analyses but were not entered into the regression models. The final analytic samples for the regression models were: parent-perpetrated DA versus IPV, *n* = 97,221; child-perpetrated DA versus IPV, *n* = 105,240; and sibling-perpetrated DA versus IPV, *n* = 98,614.

Because the dataset was anonymised analyses could not be corrected for clustering at these levels. All inferential tests were therefore conducted at the incident level.

Whilst the bivariate analyses identified variables associated with each familial DA subgroup individually, they did not account for the potential influence of other variables. Therefore, separate binary logistic regression analyses were subsequently conducted for parent-, child-, and sibling-perpetrated DA, with IPV serving as the reference category.

This allowed characteristics independently associated with each familial subgroup to be identified after controlling for all other variables included in the model. Only variables demonstrating statistically significant associations in the corresponding bivariate analyses were entered into each regression model.

While we recognise that variable selection should not be based on statistical significance alone (e.g., Heinze & Dunkler, 2017), the evidence base for relationship specific forms of familial DA remains limited, meaning there was not a clear theoretical basis for deciding in advance which police recorded variables should be prioritised for each subgroup model. Bivariate screening was therefore used as a pragmatic first step to identify candidate variables for the regression models (Bursac et al., 2008). Given this decision, the regression models are not intended to create a new frontline risk prediction tool, but to explore which routinely recorded police characteristics continue to distinguish each familial DA subgroup from IPV after adjustment for other available variables. As such, the regression findings should be interpreted as exploratory and explanatory rather than causal or predictive.

Adjusted odds ratios (ORs) and 95% confidence intervals (CIs) are reported, with statistical significance set at *p* < 0.05. Logistic regression diagnostics were conducted to assess multicollinearity, linearity in the logit for continuous age variables, and model performance. Multicollinearity was assessed for each logistic regression model using variance inflation factors (VIFs) and tolerance values. As SPSS does not provide VIF statistics directly for binary logistic regression, these diagnostics were calculated using equivalent linear regression models containing the same independent variables as each logistic regression model. VIF values below 5 and tolerance values above 0.20 were interpreted as indicating no problematic multicollinearity (Chatterjee & Simonoff, 2013; O’Brien, 2007).

The assumption of linearity in the logit for the continuous age variables was assessed using the Box–Tidwell approach, by entering interaction terms between each age variable and its natural logarithm into the corresponding logistic regression models (Box & Tidwell, 1962; Hosmer et al., 2013). Because these tests identified evidence of non-linearity, age odds ratios were reported for transparency but were not interpreted as linear year on year effects in the Results or Discussion. Model performance was assessed using classification accuracy, sensitivity, specificity, Hosmer–Lemeshow goodness-of-fit tests, and receiver operating characteristic curves with area under the curve (AUC), consistent with established approaches to evaluating logistic regression models (Hosmer et al., 2013). Sensitivity was defined as the percentage of familial DA cases correctly classified, while specificity was defined as the percentage of IPV cases correctly classified. Influential observations and residual diagnostics were not formally assessed and are therefore acknowledged as a limitation.

Bonferroni correction was applied to the bivariate analyses because these involved multiple separate comparisons across the same dataset. However, the logistic regression models were treated as adjusted explanatory models, rather than as a series of repeated standalone tests. Therefore, an additional Bonferroni correction was not applied to the regression coefficients. This approach is consistent with arguments that automatic adjustment for multiple comparisons is not always required where analyses address specific research questions (Rothman, 1990; Perneger, 1998).

Given the large sample size, findings were interpreted cautiously, with emphasis placed on effect sizes, odds ratios, confidence intervals, and consistency with the wider pattern of results, to distinguish statistically signficant differences from those likely to be of practical importance.

## 3. Results

Of the 122,276 DA cases included in the full dataset, 92,917 involved IPV. Each familial subgroup (parent-, child-, and sibling-perpetrated DA) was compared separately with IPV to examine whether perpetrator, victim, offence, DASH risk, and case outcome characteristics differed from those observed in IPV cases.

Because the IPV comparison group was identical across all three subgroups, descriptive statistics for all three comparisons are presented together in Table 1, with corresponding chi-square and Mann–Whitney U statistics presented together in Table 2.

### 3.1. Child-Perpetrated DA Compared with IPV

#### 3.1.1. Perpetrator Characteristics

As shown in Table 1 and Table 2, child perpetrators were significantly younger than IPV perpetrators (median age 25.0 vs. 34.0; Z = −79.06, *p* < 0.001, r = 0.244), a small-to-medium effect. Perpetrator gender was significantly associated with perpetrator group, although the effect size was very small (χ^2^ (1) = 45.37, *p* < 0.001, V = 0.021); child perpetrators were slightly more likely to be female (22.6%) than IPV perpetrators (20.0% female). Perpetrator ethnicity was also significantly associated with perpetrator group, although again the effect size was very small (χ^2^ (4) = 74.45, *p* < 0.001, V = 0.029); child perpetrators were more likely to be Asian (7.0% vs. 5.7%) and less likely to be Black (0.4% vs. 1.1%) than IPV perpetrators, with similar proportions of White perpetrators across both groups (91.3% vs. 92.0%).

#### 3.1.2. Victim Characteristics

As shown in Table 1 and Table 2, victims of child-perpetrated DA were significantly older than IPV victims (median age 52.0 vs. 33.0; Z = −137.74, *p* < 0.001, r = 0.425), a medium-to-large effect and the largest age-related effect observed in the present study. Victim gender was significantly associated with perpetrator group, although the effect size was very small (χ^2^ (1) = 391.46, *p* < 0.001, V = 0.061); victims of child-perpetrated abuse were more likely to be male (28.0%) than IPV victims (20.3%). Victim ethnicity was also significantly associated with perpetrator group, with a very small effect size (χ^2^ (4) = 117.15, *p* < 0.001, V = 0.035); victims of child-perpetrated abuse were more likely to be Asian (7.4% vs. 5.3%) and slightly less likely to be White (91.9% vs. 93.2%) than IPV victims.

#### 3.1.3. Offence Characteristics

As shown in Table 1 and Table 2, primary offence type showed the strongest association with perpetrator group of any categorical variable in the analysis, reaching a small-to-moderate effect size (χ^2^ (6) = 3043.30, *p* < 0.001, V = 0.202). Child-perpetrated cases were more likely to involve theft (6.9% vs. 3.8%) and arson and criminal damage (20.1% vs. 7.8%) than IPV, and less likely to involve sexual offences (0.2% vs. 3.1%), violence with injury (17.0% vs. 21.8%), and stalking and harassment (12.2% vs. 31.6%). Rates of violence without injury were higher in child-perpetrated cases (38.9% vs. 29.0%). DASH risk level was also significantly associated with perpetrator group, although the effect size was very small (χ^2^ (2) = 480.43, *p* < 0.001, V = 0.068); child-perpetrated cases were less likely to be classified as high risk (6.4% vs. 12.6%) and more likely to be classified as standard risk (53.0% vs. 45.9%) than IPV. Repeat DA was significantly less common in child-perpetrated cases than IPV, with a very small effect size (χ^2^ (1) = 512.82, *p* < 0.001, V = 0.070; 32.2% vs. 42.9%), as was online crime (χ^2^ (1) = 238.65, *p* < 0.001, V = 0.048; 1.4% vs. 4.2%) and risk involving children (χ^2^ (1) = 28.14, *p* < 0.001, V = 0.016; 4.9% vs. 6.2%). Conversely, mental health concerns were significantly higher in child-perpetrated cases than IPV, with a very small effect size (χ^2^ (1) = 877.49, *p* < 0.001, V = 0.091; 7.4% vs. 2.5%). Alcohol involvement (χ^2^ (1) = 1.26, *p* = 0.262, V = 0.003; 24.8% vs. 24.3%) and intimidation (χ^2^ (1) = 0.63, *p* = 0.429, V = 0.002; 5.5% vs. 5.3%) were not significantly associated with perpetrator group.

#### 3.1.4. Case Outcomes

As shown in Table 1 and Table 2, case outcome was significantly associated with perpetrator group, although the effect size was very small (χ^2^ (5) = 341.47, *p* < 0.001, V = 0.070). Child-perpetrated cases were more likely to result in a case outcome of victim does not support (74.1% vs. 68.2%) and resolved out of court (4.2% vs. 2.5%) than IPV, and less likely to result in evidential difficulties (8.2% vs. 14.6%) or a charge (8.4% vs. 9.8%).

### 3.2. Parent-Perpetrated DA Compared with IPV

#### 3.2.1. Perpetrator Characteristics

As shown in Table 1 and Table 2, parent perpetrators were significantly older than IPV perpetrators (median age 49.0 vs. 34.0; Z = −68.96, *p* < 0.001, r = 0.221), a small-to-medium effect. Perpetrator gender showed the strongest association with perpetrator group of any variable in the parent comparison, reaching a small-to-moderate effect size (χ^2^ (1) = 1648.33, *p* < 0.001, V = 0.130); nearly half of parent perpetrators were female (45.8%), compared to one in five IPV perpetrators (20.0%). Perpetrator ethnicity was also significantly associated with perpetrator group, although the effect size was very small (χ^2^ (4) = 123.30, *p* < 0.001, V = 0.039); parent perpetrators were more likely to be Asian (9.8% vs. 5.7%) and less likely to be Black (0.6% vs. 1.1%) or White (88.9% vs. 92.0%) than IPV perpetrators.

#### 3.2.2. Victim Characteristics

As shown in Table 1 and Table 2, victims of parent-perpetrated DA were significantly younger than IPV victims (median age 23.0 vs. 33.0; Z = −51.76, *p* < 0.001, r = 0.166), a small-to-medium effect. Victim gender was significantly associated with perpetrator group, approaching the small-to-moderate threshold (χ^2^ (1) = 892.79, *p* < 0.001, V = 0.096); victims of parent-perpetrated abuse were substantially more likely to be male (39.3%) than IPV victims (20.3%). Victim ethnicity was also significantly associated with perpetrator group, with a very small effect size (χ^2^ (4) = 149.55, *p* < 0.001, V = 0.042); victims of parent-perpetrated abuse were more likely to be Asian (9.9% vs. 5.3%) and less likely to be White (88.5% vs. 93.2%) than IPV victims.

#### 3.2.3. Offence Characteristics

As shown in Table 1 and Table 2, primary offence type was significantly associated with perpetrator group, reaching a small-to-moderate effect size (χ^2^ (6) = 764.80, *p* < 0.001, V = 0.105). Parent-perpetrated cases were more likely to involve violence without injury (49.2% vs. 29.0%) than IPV, and less likely to involve arson and criminal damage (2.5% vs. 7.8%) and stalking and harassment (19.8% vs. 31.6%). DASH risk level was also significantly associated with perpetrator group, although the effect size was very small (χ^2^ (2) = 285.60, *p* < 0.001, V = 0.054); parent-perpetrated cases were less likely to be classified as high risk (6.9% vs. 12.6%) and more likely to be classified as standard risk (58.1% vs. 45.9%) than IPV. Repeat DA was significantly less common in parent-perpetrated cases than IPV, with a very small effect size (χ^2^ (1) = 410.17, *p* < 0.001, V = 0.065; 27.3% vs. 42.9%), as was online crime (χ^2^ (1) = 23.18, *p* < 0.001, V = 0.015; 2.7% vs. 4.2%). Conversely, risk involving children (χ^2^ (1) = 143.80, *p* < 0.001, V = 0.038; 10.7% vs. 6.2%) and mental health concerns (χ^2^ (1) = 106.65, *p* < 0.001, V = 0.033; 5.0% vs. 2.5%) were significantly higher in parent-perpetrated cases, both very small effects. Alcohol involvement was not significantly associated with perpetrator group (χ^2^ (1) = 0.08, *p* = 0.774, V = 0.001; 24.5% vs. 24.3%), and intimidation did not remain significant following Bonferroni correction (χ^2^ (1) = 5.28, *p* = 0.022, V = 0.007; 4.5% vs. 5.3%).

#### 3.2.4. Case Outcomes

As shown in Table 1 and Table 2, case outcome was significantly associated with perpetrator group, although the effect size was very small (χ^2^ (5) = 156.01, *p* < 0.001, V = 0.049). Parent-perpetrated cases were more likely to result in a case outcome of victim does not support (73.6% vs. 68.2%) than IPV, and notably less likely to result in a charge (3.5% vs. 9.8%).

### 3.3. Sibling-Perpetrated DA Compared with IPV

#### 3.3.1. Perpetrator Characteristics

As shown in Table 1 and Table 2, there was a statistically significant but very small difference in perpetrator age between sibling-perpetrated DA and IPV (median age 31.0 vs. 34.0; Z = −19.02, *p* < 0.001, r = 0.061). Perpetrator gender was significantly associated with perpetrator group, with a very small effect size (χ^2^ (1) = 88.31, *p* < 0.001, V = 0.030); sibling perpetrators were more likely to be female (25.1%) than IPV perpetrators (20.0%). Perpetrator ethnicity was also significantly associated with perpetrator group, approaching the threshold for a small-to-moderate effect (χ^2^ (4) = 731.47, *p* < 0.001, V = 0.094); sibling perpetrators were substantially more likely to be Asian (15.5% vs. 5.7%) and less likely to be White (82.7% vs. 92.0%) than IPV perpetrators.

#### 3.3.2. Victim Characteristics

As shown in Table 1 and Table 2, there was a statistically significant but very small difference in victim age between sibling-perpetrated DA and IPV (median age 31.0 vs. 33.0; Z = −9.61, *p* < 0.001, r = 0.031). Victim gender showed the strongest association with perpetrator group of any variable in the sibling comparison, reaching a small-to-moderate effect size (χ^2^ (1) = 1758.21, *p* < 0.001, V = 0.134); sibling-perpetrated cases were substantially more likely than IPV cases to involve male victims (43.9% vs. 20.3%). Victim ethnicity was also significantly associated with perpetrator group, reaching a small-to-moderate effect size (χ^2^ (4) = 1097.59, *p* < 0.001, V = 0.112); sibling-perpetrated cases were substantially more likely than IPV cases to involve Asian victims (16.8% vs. 5.3%) and less likely to involve White victims (81.7% vs. 93.2%).

#### 3.3.3. Offence Characteristics

As shown in Table 1 and Table 2, repeat DA reached a small-to-moderate association with perpetrator group (χ^2^ (1) = 980.38, *p* < 0.001, V = 0.100); sibling-perpetrated cases were substantially less likely than IPV to involve repeat DA (21.9% vs. 42.9%). DASH risk level was also significantly associated with perpetrator group, approaching the threshold for a small-to-moderate effect (χ^2^ (2) = 851.18, *p* < 0.001, V = 0.093); sibling-perpetrated cases were less likely to be classified as high risk (4.3% vs. 12.6%) and more likely to be classified as standard risk (64.6% vs. 45.9%) than IPV. Primary offence type was significantly associated with perpetrator group, with a very small effect size (χ^2^ (6) = 424.57, *p* < 0.001, V = 0.077); sibling-perpetrated cases were more likely to involve violence with injury (25.3% vs. 21.8%) and violence without injury (37.6% vs. 29.0%), and less likely to involve stalking and harassment (21.2% vs. 31.6%) and sexual offences (0.4% vs. 3.1%), than IPV. Intimidation was significantly higher in sibling-perpetrated cases than IPV, with a very small effect size (χ^2^ (1) = 25.20, *p* < 0.001, V = 0.016; 6.8% vs. 5.3%), as were mental health concerns (χ^2^ (1) = 138.80, *p* < 0.001, V = 0.038; 5.1% vs. 2.5%), while risk involving children was significantly lower (χ^2^ (1) = 66.35, *p* < 0.001, V = 0.026; 3.5% vs. 6.2%). Alcohol involvement (χ^2^ (1) = 1.46, *p* = 0.228, V = 0.004; 23.6% vs. 24.3%) and online crime (χ^2^ (1) = 0.48, *p* = 0.488, V = 0.002; 4.4% vs. 4.2%) were not significantly associated with perpetrator group.

#### 3.3.4. Case Outcomes

As shown in Table 1 and Table 2, case outcome was significantly associated with perpetrator group, although the effect size was very small (χ^2^ (5) = 198.49, *p* < 0.001, V = 0.055). Sibling-perpetrated cases were more likely to result in a case outcome of victim does not support (75.0% vs. 68.2%) than IPV, and less likely to result in a charge (4.3% vs. 9.8%) or evidential difficulties (12.3% vs. 14.6%).

### 3.4. Binary Logistic Regression Across All Three Subgroups

Binary logistic regression was conducted separately for each subgroup to identify characteristics independently associated with subgroup membership compared with IPV, after accounting for other variables in each model. Results for all three subgroups are presented together in Table 3. Where adjusted odds ratios differ in direction from the bivariate findings, this reflects adjustment for correlated predictors within the multivariable model rather than contradictory findings.

Model diagnostics and performance were examined before interpreting the subgroup specific regression findings. Multicollinearity diagnostics did not indicate problematic collinearity among predictors. Across the three models, VIF values ranged from 1.00 to 2.96 and tolerance values ranged from 0.338 to 0.996. VIFs ranged from 1.01 to 2.29 in the child-perpetrated model, 1.00 to 2.96 in the parent perpetrated model, and 1.00 to 2.86 in the sibling-perpetrated model. These values suggest that the observed direction changes for some gender variables are unlikely to reflect coefficient instability caused by multicollinearity (Chatterjee & Simonoff, 2013). Non-age coefficients are therefore interpreted as adjusted, conditional associations, showing how each predictor distinguished the familial DA subgroup from IPV after accounting for the other variables in the model.

The assumption of linearity in the logit was assessed for perpetrator age and victim age using the Box–Tidwell approach. The age-by-log-age interaction terms were statistically significant across the models, indicating evidence of non-linearity in the relationship between age and subgroup membership. Age should therefore not be interpreted as a strictly linear predictor in these models. Age ORs are reported in Table 3 for transparency only and are not interpreted below as linear year-on-year changes in odds. Instead, age is interpreted directionally, in terms of whether familial DA cases involved younger or older perpetrators or victims than IPV.

Model performance was also assessed using Hosmer–Lemeshow goodness-of-fit tests, classification accuracy, sensitivity, specificity, and ROC/AUC values. The child-perpetrated model showed excellent discrimination, with an AUC of 0.985, overall classification accuracy of 98.2%, sensitivity of 92.8%, and specificity of 98.9%. The parent-perpetrated model also showed strong discrimination, with an AUC of 0.930, overall accuracy of 98.1%, sensitivity of 67.1%, and specificity of 99.5%. Hosmer–Lemeshow tests were significant across all three models: child, χ^2^ (8) = 12,191.00, *p* < 0.001; parent, χ^2^ (8) = 13,188.35, *p* < 0.001; and sibling, χ^2^ (8) = 4077.76, *p* < 0.001. Given the very large sample size, this calibration result should be interpreted cautiously (Paul et al., 2013). The sibling-perpetrated model showed more limited performance, with a low Nagelkerke R^2^ (0.108), weaker discrimination (AUC = 0.661), high overall accuracy (94.3%), very low sensitivity (1.0%), and specificity of 100.0%. This pattern indicates that the sibling model largely classified cases as IPV at the default threshold. This does not automatically invalidate individual adjusted associations, but it does mean that sibling-model coefficients should be treated as descriptive conditional relationships within the estimated model, rather than as discriminative predictors with practical utility.

#### 3.4.1. Child-Perpetrated DA

The child-perpetrated model produced a very high Nagelkerke R^2^ (Nagelkerke R^2^ = 0.853) To examine why this was, an additional hierarchical logistic regression was conducted and showed that this was mainly explained by age differences between child-perpetrated DA and IPV. When perpetrator and victim age were entered alone, the model produced a Nagelkerke R^2^ of 0.841. Adding the remaining predictors increased this only slightly to 0.853, ΔR^2^ = 0.012, although this change was statistically significant, χ^2^ (6) = 1175.12, *p* < 0.001. Because the Box–Tidwell tests indicated that the relationship between age and subgroup membership was not strictly linear, these findings are interpreted as showing a broad age pattern rather than a precise year-by-year age effect. In practical terms, child-perpetrated DA generally involved younger perpetrators and older victims than IPV. Primary offence type produced the largest bivariate effect size in the analysis (V = 0.202), with child-perpetrated cases markedly more likely to involve arson and criminal damage and theft, and less likely to involve stalking, sexual offences, or violence with injury, than IPV. Mental health concerns remained independently associated with child-perpetrated DA in the adjusted model (OR = 1.853), consistent with the elevated bivariate rate (7.4% vs. 2.5%; V = 0.091). Although child-perpetrated cases were more likely to involve male victims at the bivariate level (28.0% vs. 20.3%; V = 0.061), female victim gender was associated with increased odds of child-perpetrated DA in the adjusted model (OR = 3.142), suggesting that the relationship between victim gender and subgroup membership are more likely to change once age and other variables were controlled for. Perpetrator gender also remained a significant predictor, with female perpetrator gender associated with lower odds of child-perpetrated DA once other variables were controlled for (OR = 0.470), despite child perpetrators being slightly more likely to be female at the bivariate level (22.6% vs. 20.0%; V = 0.021). Repeat DA remained a significant predictor in the protective direction (OR = 0.631; bivariate 32.2% vs. 42.9%, V = 0.070), and Online crime also remained statistically significant in the adjusted model (OR = 0.675), whereas risk involving children did not remain significant in the full model. Only 6.4% of child-perpetrated cases were classified as high risk on DASH, compared with 12.6% of IPV cases.

#### 3.4.2. Parent-Perpetrated DA

The parent-perpetrated model demonstrated strong explanatory value (Nagelkerke R^2^ = 0.623). Parent-perpetrated DA was most clearly distinguished from IPV by perpetrator gender at the bivariate level (V = 0.130; 45.8% of parent perpetrators were female compared with 20.0% of IPV perpetrators), and perpetrator gender was the strongest independent predictor in the regression model (OR = 5.078). Parent-perpetrated DA showed a reversed age pattern compared with IPV, with older perpetrators and younger victims. Because age did not have a strictly linear relationship with subgroup membership, this should be interpreted as a broad age profile rather than as a precise year by year effect.

Primary offence type reached a small-to-moderate bivariate effect size (V = 0.105), with parent-perpetrated cases more likely to involve violence without injury (49.2% vs. 29.0%) and less likely to involve stalking and harassment (19.8% vs. 31.6%) than IPV. Risk involving children (OR = 2.235; bivariate 10.7% vs. 6.2%, V = 0.038) and mental health concerns (OR = 1.673; bivariate 5.0% vs. 2.5%, V = 0.033) were also independently associated with parent-perpetrated DA. Online crime also remained statistically significant in the parent model (OR = 0.734), although the effect was modest. Alcohol involvement was non-significant, and intimidation did not survive Bonferroni correction. Only 6.9% of parent-perpetrated cases were classified as high risk on DASH, compared with 12.6% of IPV cases.

#### 3.4.3. Sibling-Perpetrated DA

The sibling-perpetrated model demonstrated modest explanatory value (Nagelkerke R^2^ = 0.108). Given the model’s low Nagelkerke R^2^, weaker AUC, and very low sensitivity, these findings should be used to understand possible patterns in the data rather than to predict or identify sibling-perpetrated DA in practice. The bivariate finding showed a substantially reduced rate of repeat DA relative to IPV (21.9% vs. 42.9%; V = 0.100), and repeat DA remained one of the strongest independent predictors in the adjusted model (OR = 0.390). Victim gender (V = 0.134) and victim ethnicity (V = 0.112) both reached small-to-moderate bivariate effect sizes, with sibling-perpetrated cases more likely than IPV to involve male victims (43.9% vs. 20.3%) and Asian victims (16.8% vs. 5.3%). Within the exploratory regression model, victim gender showed the largest adjusted association with sibling-perpetrated DA (OR = 0.139).Perpetrator gender was also independently associated with sibling-perpetrated DA (OR = 0.242), although the direction of this association differed from the bivariate comparison, in which sibling perpetrators were descriptively more likely to be female than IPV perpetrators (25.1% vs. 20.0%; V = 0.030); this suggests the relationship between perpetrator gender and subgroup membership changed once other variables were controlled for. Mental health concerns (OR = 2.157; bivariate 5.1% vs. 2.5%, V = 0.038) and intimidation (OR = 1.787; bivariate 6.8% vs. 5.3%, V = 0.016) were also independently associated with sibling-perpetrated cases, while risk involving children was significantly less likely than in IPV (OR = 0.667; bivariate 3.5% vs. 6.2%, V = 0.026). Unlike child- and parent-perpetrated cases, sibling- perpetrated cases showed only minimal age differences from IPV. In line with the non-linearity identified, these age findings are interpreted only as broad directional patterns, rather than as precise year-by-year effects.

## 4. Discussion

The current study examined whether parent-, child-, and sibling-perpetrated DA differed from IPV in perpetrator, victim, offence, DASH risk, and case outcome characteristics. Rather than treating familial DA as one broad category, the study compared each familial perpetrator relationship separately with IPV. The key contribution is therefore not simply that familial DA differs from IPV, but that these differences were not consistent across familial relationships.

Overall, child-perpetrated DA showed the clearest observed difference from IPV, particularly in relation to age profile and offence type. Parent-perpetrated DA showed a more selective pattern of differences, most notably in relation to perpetrator gender, age profile, and offence type. Sibling-perpetrated DA showed a different pattern again, with the clearest differences relating to victim gender, victim ethnicity, and repeat DA. However, because the regression model for sibling-perpetrated DA had weaker overall performance, these findings interpreted cautiously. Together, the findings suggest that practitioners should not assume that all familial DA cases will look the same, or that they will necessarily mirror IPV. Different family relationships may involve different patterns of risk, harm, reporting, and support need.

### 4.1. Child-Perpetrated DA

Child-perpetrated DA showed the clearest observed divergence from IPV. The most notable difference related to age. Child perpetrators were substantially younger than IPV perpetrators, while victims were substantially older than IPV victims. This is unsurprising given the relational structure of child-to-parent or child-to-family abuse, but it is still important for practice. It demonstrates that child-perpetrated DA has a different age profile to IPV and therefore may involve different relational dynamics, forms of dependency, and barriers to disclosure. It suggests that child-perpetrated DA may involve different family dynamics, forms of dependency, and barriers to disclosure than those usually associated with IPV. The hierarchical regression indicated that age accounted for most of the model fit in the child-perpetrated comparison. However, because the relationship between age and subgroup membership was not strictly linear, this should be understood as a broad age pattern rather than a precise year by year effect. In practical terms, child-perpetrated DA generally involved younger perpetrators and older victims than IPV, but this finding should not be treated as a tool for predicting or identifying child-perpetrated DA in frontline practice.

Child-perpetrated DA also differed from IPV in offence type. These cases were more likely to involve arson and criminal damage, theft, and violence without injury, and less likely to involve stalking and harassment, sexual offences, or violence with injury. This suggests that child-perpetrated DA may involve a different pattern of harm to IPV. Rather than being characterised by the offence types more commonly associated with intimate partner abuse, such as stalking and harassment, child-perpetrated DA may be more likely to involve conflict within the home, damage to property, or financially motivated incidents. This fits with existing literature which highlights the role of family conflict, dependency, and household strain in child-to-parent abuse (Cottrell, 2001; A. Holt, 2016; McManus et al., 2017).

Mental health concerns were also more commonly recorded in child-perpetrated DA than IPV and remained associated with child-perpetrated cases in the regression model. This supports existing research suggesting that child-to-parent abuse is often linked with complex individual and family circumstances, including emotional, behavioural, and mental health difficulties (McManus et al., 2017). However, this finding should be interpreted cautiously. The current study used police-recorded mental health concerns, not clinical diagnoses. Therefore, the finding does not show that mental health causes child-perpetrated DA, but rather that mental health concerns appear more frequently in police recorded child-perpetrated cases than in IPV cases.

Interestingly, child-perpetrated DA was less likely than IPV to be classified as high risk on DASH. Only 6.4% of child-perpetrated cases were classified as high risk, compared with 12.6% of IPV cases. On one hand, this may indicate that child-perpetrated cases are less likely to contain the specific risk indicators captured by DASH. On the other hand, it may indicate that DASH does not fully capture the types of risk present in child-perpetrated DA. This is particularly important because parents may be reluctant to report or support criminal justice action against their child due to shame, guilt, fear of family disruption, or the child’s reliance on them (Brule & Eckstein, 2016; Cottrell, 2001; A. Holt, 2016). Therefore, lower high-risk classification should not automatically be interpreted as lower need or lower harm.

This finding is also important given wider social changes. As more adult children remain living with parents for longer, often because of financial pressures and housing difficulties, there may be increased opportunities for conflict and dependency within the home. These circumstances may shape both the form of abuse and the victim’s ability to seek help. Therefore, child-perpetrated DA may require greater visibility within policing and support services, alongside risk assessment approaches that recognise the specific dynamics of parent-child relationships.

### 4.2. Parent-Perpetrated DA

Parent-perpetrated DA also differed from IPV, although the differences were more selective. The clearest difference related to perpetrator gender. Nearly half of parent perpetrators were female, compared with one fifth of IPV perpetrators. This finding is important because it challenges the assumption that DA cases will necessarily follow a male perpetrator/female victim intimate partner pattern. While this assumption is grounded in the gendered nature of IPV, it may be less applicable to some forms of familial DA.

The gender profile of parent-perpetrated DA may reflect the different social and care giving roles involved in parent-child relationships. Parents, including mothers, may remain closely connected to adult children through caregiving, financial support, co-residence, or ongoing family responsibility. These dynamics differ from IPV and may shape both the nature of abuse and the way it is reported. However, this should not be interpreted as suggesting that parent-perpetrated DA is less serious. Rather, it shows that the demographic profile of perpetrators and victims can differ substantially depending on the relationship context.

Parent-perpetrated DA also showed a reversed age pattern compared with IPV, with older perpetrators and younger victims. Because age did not have a strictly linear relationship with subgroup membership, this should be interpreted as a broad age profile rather than as a precise year by year effect. For practitioners, this matters because a younger victim and older perpetrator may indicate a very different family context from that usually seen in IPV. Police and support services should therefore avoid relying too heavily on IPV-informed assumptions when identifying risk, vulnerability, or support needs in familial DA cases.

In terms of offence characteristics, parent-perpetrated DA was more likely than IPV to involve violence without injury and less likely to involve stalking and harassment. This may suggest that parent-perpetrated DA is less likely to involve some offence patterns commonly associated with IPV, and more likely to involve physical conflict within continuing family relationships. Mental health concerns and risk involving children were also independently associated with parent-perpetrated DA, which aligns with existing research linking parent-to-child abuse with parental mental health difficulties and wider family vulnerability (Augusti et al., 2021; David-Ferdon, 2021; Patwardhan et al., 2017). However, as with the child-perpetrated findings, these associations were small and based on police-recorded indicators.

### 4.3. Sibling-Perpetrated DA

Sibling-perpetrated DA showed a different pattern again. Unlike child- and parent-perpetrated DA, sibling-perpetrated DA showed very little age difference from IPV. The strongest differences related instead to victim gender, victim ethnicity, and repeat DA. Sibling-perpetrated cases were more likely than IPV to involve male victims and Asian victims, and were substantially less likely to involve repeat DA. Because the sibling-perpetrated model had weaker overall performance, these findings should be treated as possible patterns in police-recorded sibling DA, rather than as factors that could reliably identify sibling-perpetrated cases in practice.

The higher proportion of male victims in sibling-perpetrated DA is particularly important. DA services and risk frameworks have historically been shaped around IPV, where female victimisation is central. While this is essential for understanding IPV, it may mean that male victims in familial DA contexts are less visible. This is especially relevant for sibling abuse, which is often minimised or dismissed as ordinary sibling conflict or rivalry (Kettrey & Emery, 2006). The current findings therefore suggest that sibling-perpetrated DA may be at risk of being overlooked unless practitioners are alert to the ways it differs from IPV.

Sibling-perpetrated DA was also less likely than IPV to involve repeat DA. This finding should be interpreted carefully. It may mean that sibling-perpetrated cases are genuinely less likely to involve repeated incidents. However, it may also reflect differences in recognition, reporting, or recording. For example, repeated sibling conflict may not always be identified as DA by victims, families, or professionals. Alternatively, families may be less likely to report multiple incidents if they perceive the abuse as private family conflict. Further research is needed to determine whether the lower repeat DA rate reflects a genuine difference in offending patterns or a difference in how sibling abuse is reported and recorded.

Mental health concerns were also more common in sibling-perpetrated DA than IPV and remained independently associated with sibling-perpetrated cases within the exploratory regression model. This is broadly consistent with previous research suggesting links between mental health difficulties and sibling aggression or abuse (Tompsett et al., 2018). Psychiatric symptoms may increase aggression directly in some cases (Girasek et al., 2022), but mental health concerns may also sit within broader family contexts involving stress, conflict, previous victimisation, or exposure to violence. Therefore, as with the other subgroups, mental health should be understood as part of a wider safeguarding picture rather than as a simple cause of abuse.

### 4.4. Similarities Across Familial DA Subgroups

Across all three familial subgroups, three findings were particularly important. First, alcohol involvement did not significantly differ between familial DA and IPV. In practical terms, alcohol appeared at similar rates across child-, parent-, sibling-perpetrated DA and IPV cases. This is important because alcohol is one of the factors commonly considered during DASH risk assessment. This suggests that alcohol may be less useful for differentiating familial DA from IPV than other factors such as age dynamics, relationship type, offence pattern, and mental health concerns. For practitioners, risk assessment in familial DA may need to look beyond standard incident-based risk indicators and pay closer attention to the specific family relationship and context in which the abuse is occurring.

Second, mental health concerns were more commonly recorded in familial DA cases than in IPV cases. This was found across all three groups: child-, parent-, and sibling-perpetrated DA. Although the bivariate effect sizes were small, the consistency of this finding is notable. This suggests that mental health concerns may be an important factor for practitioners to consider when responding to familial DA. This does not mean that mental health difficulties cause familial abuse. Rather, it highlights the need for police and support services to consider whether mental health concerns are present and how they may affect risk, safeguarding needs, and the type of support required.

Third, all three familial subgroups were less likely than IPV to be classified as high risk on DASH. It may indicate that familial DA cases are less likely to contain the forms of risk captured by DASH. However, it may also suggest that DASH, as an IPV informed tool, may be less sensitive to the risks present in familial DA. This is particularly relevant given existing concerns about the predictive validity of DASH and its ability to identify the most vulnerable victims (Almond et al., 2017; McManus & Phoenix, 2023; Turner et al., 2019, 2022). The current findings therefore raise questions about whether any standard DA risk assessment approach can adequately capture risk across all DA relationship contexts.

### 4.5. Implications

Findings from the current study suggest that parent-, child-, and sibling-perpetrated DA should not be treated as interchangeable forms of familial DA. The observed patterns differed from IPV in different ways and to different degrees, meaning that risk assessment, frontline policing responses, safeguarding decisions, and support pathways may need to be more explicitly relationship-informed. These implications are drawn from exploratory, incident-level patterns rather than from a validated predictive model.

This is also important as policing moves towards newer approaches such as the Domestic Abuse Risk Assessment (DARA) (College of Policing, 2022). Although the current study necessarily focuses on DASH because this was the tool embedded in the dataset, the findings have clear implications for DARA implementation and evaluation. Replacing one assessment tool with another will not, in itself, address the issue if newer approaches are validated predominantly against IPV populations or implemented without attention to different forms of familial DA.

Across all three familial subgroups, cases were less likely than IPV to be classified as high risk, despite showing distinct patterns of harm, including different age dynamics, offence types, mental health concerns, and victim/perpetrator profiles. This does not mean that DASH is irrelevant or should not be used in familial DA cases. However, it does suggest that DASH should not be relied upon as a one-size-fits-all tool without considering the specific family relationship and context in which the abuse is occurring.

For practice, the findings point to the need for officer training and supervision that helps practitioners recognise relationship-specific dynamics. This may be particularly important in child-perpetrated DA, where co-residence, dependency, parental shame, mental health concerns, property damage, and reluctance to support criminal justice action may shape both risk and engagement. These issues may not be fully reflected in IPV-informed risk indicators.

At a service level, the findings also have implications for safeguarding pathways and commissioning. Specialist responses may need to account for different family relationships rather than treating familial DA as a single category, with clearer routes into mental health support, family-based safeguarding, adult and child safeguarding, and tailored perpetrator and victim support where appropriate.

Overall, police and support services may need to supplement standard DA risk assessment with relationship-specific professional judgement, supplementary prompts, or validated add-on tools for familial DA. This would help ensure that risk, harm, and safeguarding needs are not underestimated simply because familial DA does not present in the same way as IPV.

### 4.6. Limitations and Future Research

Limitations of the current study predominantly centre around the use of police recorded data. Critically, only reported and recorded DA cases were investigated in this study which may have potentially impacted the degree of difference noted between the different perpetrator sub-types. According to the crime survey 2025 3.8 million people were subject to DA in the last year yet during the same period the police recorded 1.4 million incidents and crimes, evidence of the scale of DA underreporting (Office for National Statistics, 2025). Familial DA may be particularly underreported due to shame, stigma, dependency, fear of family disruption, or reluctance to criminalise a family member. Patterns of family violence disclosure, help-seeking, and intergenerational transmission may also vary across cultural contexts. DA generally As perpetrator, victim, offence, and outcome characteristics may differ between reported and unreported cases, findings may not generalise to all DA cases.

A further limitation is that the analyses were conducted at incident level. The anonymised dataset did not include reliable victim, perpetrator, or perpetrator-victim dyad identifiers. It is therefore possible that the same victim, perpetrator, or dyad contributed more than one incident to the dataset. This means that the reported *p* values and confidence intervals should be treated as approximate indicators of uncertainty. If repeated incidents involving the same individuals or dyads were present, the statistical tests may underestimate uncertainty by producing narrower confidence intervals or smaller *p* values than would have been obtained if clustering could have been modelled. For this reason, the findings have been interpreted cautiously, with emphasis placed on effect sizes, confidence intervals, and the practical pattern of results. Future research should use clustered or longitudinal approaches where suitable identifiers are available.

Similarly, in a substantial number of cases, the ethnicity information was not recorded. This may have resulted in an increased proportion of White individuals this has implications for understanding some of the subtle distinctions between different forms of DA. Future research should examine ethnicity and geographical origin in more detail to explore if DA perpetrators present differently given that patterns of family violence disclosure, help seeing and intergenerational transmission may vary substantially from Anglo-American contexts.

Likewise, only cases within a single police force were investigated. Given geographical differences in DA prevalence, demographics, recording practices, and service availability, future research should examine whether these subgroup differences replicate across additional police force areas (Office for National Statistics, 2025; Peek-Asa et al., 2011; Garcia-Moreno et al., 2006).

A further limitation concerns the interpretation of the regression models. The very high Nagelkerke R^2^ in the child-perpetrated model is likely to reflect the structurally different age profiles inherent in parent-child relationships, compared with IPV, rather than indicating that the model has practical predictive value. In addition, Box–Tidwell tests indicated that age did not have a strictly linear relationship with subgroup membership. Age related findings should therefore be interpreted as broad age patterns, rather than as precise year on year effects. The sibling-perpetrated regression model also had limited explanatory and discriminatory performance. Its findings should therefore be interpreted as descriptive patterns within police-recorded data, rather than as evidence that the model could reliably distinguish sibling-perpetrated DA from IPV in practice.

The relationship categories used in the current study were also necessarily broad. Parent-, child-, and sibling-perpetrated DA may each include a range of circumstances, including biological, step, adoptive, and blended family relationships, as well as variation in co-residence, dependency, caregiving, and financial arrangements. These contextual details were not available in the current dataset. The IPV sample was also broad containing both current partner and ex partner, it may be the case that these two subgroups differ something which should be explored in future research.

Finally, the current study only included cases meeting the legal definition of DA, meaning that victims and perpetrators were aged 16 or over. This is important because much of the existing literature on child-to-parent abuse includes younger adolescents. The findings therefore relate specifically to police-recorded DA involving individuals aged 16 or over and may not apply to younger adolescent-to-parent violence. Future research should also consider how cases of physical DA may differ from those more centred around psychological violence and coercive control.

## 5. Conclusions

Overall, the current study found that parent-, child-, and sibling-perpetrated DA showed different observed patterns when compared separately with IPV. Child-perpetrated DA showed the clearest divergence from IPV, particularly in relation to age dynamics and offence type. Parent-perpetrated DA was most clearly distinguished by perpetrator gender, age dynamics, and offence type, while sibling-perpetrated DA was most clearly distinguished by victim gender, victim ethnicity, and lower repeat DA. Across all three familial subgroups, mental health concerns were more commonly recorded than in IPV, while high-risk DASH classifications were less common.

The central contribution is that familial DA does not simply differ from IPV as a broad category; rather, parent-, child-, and sibling-perpetrated DA each presented differently. While IPV-informed responses remain essential, current risk assessment, policing, perpetrator intervention, and victim support pathways may need to be adapted to better recognise relationship-specific dynamics within familial DA. This may be particularly important for child-perpetrated DA, where the findings suggest that current responses may not fully capture the nature of risk, harm, and support needs. Future research should examine whether relationship-specific assessment frameworks, or validated supplementary tools, are required to support risk assessment and safeguarding across different forms of familial DA.

## Figures and Tables

**Table 1:** Perpetrator, Victim, Offence, DASH Risk, and Case Outcome Characteristics for Parent-, Child-, Sibling and IPV Perpetrated DA.

	Perpetrator Group			
Variable	Parent, *n* (%)	Child, *n* (%)	Sibling, *n* (%)	IPV, *n* (%)
*n*	4304	12,323	5697	92,917
Perpetrator characteristics				
Median age (IQR)	49.0 (15.0)	25.0 (14.0)	31.0 (16.0)	34.0 (14.0)
Gender (*n* = 115,241)				
Male	2332 (54.2)	9541 (77.4)	4265 (74.9)	74,353 (80.0)
Female	1972 (45.8)	2782 (22.6)	1432 (25.1)	18,564 (20.0)
Ethnicity (*n* = 96,230)				
Black	20 (0.6)	46 (0.4)	26 (0.6)	837 (1.1)
Asian	337 (9.8)	767 (7.0)	721 (15.5)	4400 (5.7)
Mixed	11 (0.3)	119 (1.1)	42 (0.9)	757 (1.0)
Other	14 (0.4)	18 (0.2)	19 (0.4)	243 (0.3)
White	3049 (88.9)	9725 (91.3)	3850 (82.7)	71,255 (92.0)
Victim characteristics				
Median age (IQR)	23.0 (13.0)	52.0 (16.0)	31.0 (16.0)	33.0 (14.0)
Gender (*n* = 115,241)				
Male	1692 (39.3)	3456 (28.0)	2499 (43.9)	18,855 (20.3)
Female	2612 (60.7)	8867 (72.0)	3198 (56.1)	74,062 (79.7)
Ethnicity (*n* = 102,484)				
Black	24 (0.6)	53 (0.5)	40 (0.8)	623 (0.8)
Asian	373 (9.9)	810 (7.4)	833 (16.8)	4410 (5.3)
Mixed	23 (0.6)	20 (0.2)	19 (0.4)	366 (0.4)
Other	14 (0.4)	7 (0.1)	16 (0.3)	211 (0.3)
White	3337 (88.5)	10,057 (91.9)	4055 (81.7)	77,193 (93.2)
Offence characteristics				
Primary offence (*n* = 83,027)				
Sexual	48 (1.4)	13 (0.2)	21 (0.4)	2075 (3.1)
Theft	76 (2.2)	590 (6.9)	145 (3.1)	2509 (3.8)
Arson and criminal damage	85 (2.5)	1715 (20.1)	396 (8.4)	5192 (7.8)
Other	115 (3.4)	404 (4.7)	186 (3.9)	1881 (2.8)
Violence with injury	729 (21.4)	1451 (17.0)	1198 (25.3)	14,486 (21.8)
Violence without injury	1674 (49.2)	3315 (38.9)	1781 (37.6)	19,240 (29.0)
Stalking and harassment	673 (19.8)	1040 (12.2)	1004 (21.2)	20,985 (31.6)
DASH risk level (*n* = 115,240)				
Standard	2502 (58.1)	6535 (53.0)	3681 (64.6)	42,664 (45.9)
Medium	1491 (34.6)	4987 (40.5)	1752 (30.8)	38,318 (41.2)
High	297 (6.9)	783 (6.4)	246 (4.3)	11,746 (12.6)
Other offence characteristics				
Repeat DA	1176 (27.3)	3968 (32.2)	1247 (21.9)	39,862 (42.9)
Alcohol	1054 (24.5)	3056 (24.8)	1344 (23.6)	22,599 (24.3)
Risk involving children	461 (10.7)	604 (4.9)	199 (3.5)	5761 (6.2)
Intimidation	194 (4.5)	678 (5.5)	387 (6.8)	4920 (5.3)
Online crime	116 (2.7)	172 (1.4)	251 (4.4)	3902 (4.2)
Mental health concerns	215 (5.0)	908 (7.4)	291 (5.1)	2306 (2.5)
Case outcome (*n* = 77,370)				
Charge	110 (3.5)	682 (8.4)	194 (4.3)	6030 (9.8)
Resolved out of court	93 (2.9)	341 (4.2)	161 (3.6)	1560 (2.5)
Evidential difficulties	437 (13.7)	667 (8.2)	552 (12.3)	8975 (14.6)
Victim does not support	2342 (73.6)	5994 (74.1)	3376 (75.0)	41,992 (68.2)
No suspect	12 (0.4)	8 (0.1)	19 (0.4)	172 (0.3)
Other	189 (5.9)	401 (5.0)	201 (4.5)	2862 (4.6)

Note. IQR = interquartile range.

**Table 2:** Chi-Square and Mann–Whitney U Results: Parent-, Child-, and Sibling-Perpetrated DA, Each Compared with the Same IPV Comparison Group.

	Perpetrator Group		
Variable	Parent vs. IPV	Child vs. IPV	Sibling vs. IPV
Perpetrator age (M-W U)	Z = −68.96, r = 0.221 *	Z = −79.06, r = 0.244 *	Z = −19.02, r = 0.061 *
Perpetrator gender	χ^2^ (1) = 1648.33, V = 0.130 *	χ^2^ (1) = 45.37, V = 0.021 *	χ^2^ (1) = 88.31, V = 0.030 *
Perpetrator ethnicity	χ^2^ (4) = 123.30, V = 0.039 *	χ^2^ (4) = 74.45, V = 0.029 *	χ^2^ (4) = 731.47, V = 0.094 *
Victim age (M-W U)	Z = −51.76, r = 0.166 *	Z = −137.74, r = 0.425 *	Z = −9.61, r = 0.031 *
Victim gender	χ^2^ (1) = 892.79, V = 0.096 *	χ^2^ (1) = 391.46, V = 0.061 *	χ^2^ (1) = 1758.21, V = 0.134 *
Victim ethnicity	χ^2^ (4) = 149.55, V = 0.042 *	χ^2^ (4) = 117.15, V = 0.035 *	χ^2^ (4) = 1097.59, V = 0.112 *
Primary offence	χ^2^ (6) = 764.80, V = 0.105 *	χ^2^ (6) = 3043.30, V = 0.202 *	χ^2^ (6) = 424.57, V = 0.077 *
DASH risk level	χ^2^ (2) = 285.60, V = 0.054 *	χ^2^ (2) = 480.43, V = 0.068 *	χ^2^ (2) = 851.18, V = 0.093 *
Repeat DA	χ^2^ (1) = 410.17, V = 0.065 *	χ^2^ (1) = 512.82, V = 0.070 *	χ^2^ (1) = 980.38, V = 0.100 *
Alcohol	χ^2^ (1) = 0.08, V = 0.001, ns	χ^2^ (1) = 1.26, V = 0.003, ns	χ^2^ (1) = 1.46, V = 0.004, ns
Risk involving children	χ^2^ (1) = 143.80, V = 0.038 *	χ^2^ (1) = 28.14, V = 0.016 *	χ^2^ (1) = 66.35, V = 0.026 *
Intimidation	χ^2^ (1) = 5.28, V = 0.007, ns	χ^2^ (1) = 0.63, V = 0.002, ns	χ^2^ (1) = 25.20, V = 0.016 *
Online crime	χ^2^ (1) = 23.18, V = 0.015 *	χ^2^ (1) = 238.65, V = 0.048 *	χ^2^ (1) = 0.48, V = 0.002, ns
Mental health concerns	χ^2^ (1) = 106.65, V = 0.033 *	χ^2^ (1) = 877.49, V = 0.091 *	χ^2^ (1) = 138.80, V = 0.038 *
Case outcome	χ^2^ (5) = 156.01, V = 0.049 *	χ^2^ (5) = 341.47, V = 0.070 *	χ^2^ (5) = 198.49, V = 0.055 *

Note. M-W U = Mann-Whitney U. * indicates significance after Bonferroni correction (*p* < 0.003); ns = not significant. V = Cramér’s V; r = Mann–Whitney U effect size.

**Table 3:** Binary Logistic Regression Predicting Parent-, Child-, and Sibling-Perpetrated DA Compared with IPV.

	Perpetrator Group		
Variable	Parent OR [95% CI]	Child OR [95% CI]	Sibling OR [95% CI]
Repeat DA	0.613 [0.556, 0.676] ***	0.631 [0.577, 0.690] ***	0.390 [0.365, 0.417] ***
Risk involving children	2.235 [1.905, 2.622] ***	1.162 [0.954, 1.415] ns	0.667 [0.576, 0.772] ***
Intimidation	—	—	1.787 [1.597, 2.000] ***
Online crime	0.734 [0.573, 0.940] *	0.675 [0.502, 0.908] **	—
Mental health concerns	1.673 [1.318, 2.124] ***	1.853 [1.499, 2.292] ***	2.157 [1.890, 2.461] ***
Victim gender	0.552 [0.494, 0.616] ***	3.142 [2.821, 3.500] ***	0.139 [0.128, 0.150] ***
Perpetrator gender	5.078 [4.549, 5.668] ***	0.470 [0.422, 0.522] ***	0.242 [0.222, 0.264] ***
Perpetrator age	1.251 [1.245, 1.257] ***	0.688 [0.683, 0.693] ***	0.972 [0.968, 0.976] ***
Victim age	0.781 [0.776, 0.786] ***	1.403 [1.394, 1.412] ***	1.008 [1.004, 1.012] ***

Note. OR = odds ratio [95% CI]. * *p* < 0.05, ** *p* < 0.01, *** *p* < 0.001; ns = not significant—indicates the variable was not significant in the bivariate analysis for that subgroup and was therefore not entered into that subgroup’s model. Age variables were entered as continuous predictors in years, however Box-Tidwell tests indicated non-linearity, so age ORs are reported for transparency and interpreted directionally rather than as year on year effects. Gender variables coded 0 = male, 1 = female; an OR above 1 indicates greater odds of the case belonging to that subgroup when the perpetrator/victim is female, and an OR below 1 indicates lower odds. Model fit: parent Nagelkerke R^2^ = 0.623; child Nagelkerke R^2^ = 0.853; sibling Nagelkerke R^2^ = 0.108. Because the data are incident-level and clustering could not be modelled, confidence intervals and *p* values should be interpreted as approximate and may under-state uncertainty.

## Data Availability

Requests to access the data must be submitted to the Police force who granted access to the researchers.

## Abbreviations

The following abbreviations are used in this manuscript:

DA	Domestic Abuse
IPV	Intimate partner violence
DASH	Domestic Abuse, Stalking, Harassment and Honour-Based Violence
